# High TMPRSS11D protein expression predicts poor overall survival in non-small cell lung cancer

**DOI:** 10.18632/oncotarget.14559

**Published:** 2017-01-09

**Authors:** Xiang Cao, Zhiyuan Tang, Fang Huang, Qin Jin, Xiaoyu Zhou, Jiahai Shi

**Affiliations:** ^1^ Department of Cardiothoracic Surgery, Nantong University Affiliated Hospital, Nantong, Jiangsu 226001, China; ^2^ Department of Respiratory Medicine, Nantong University Affiliated Hospital, Nantong, Jiangsu 226001, China; ^3^ Department of Pathology, Nantong University Affiliated Hospital, Nantong, Jiangsu 226001, China

**Keywords:** TMPRSS11D, non-small cell lung cancer, immunohistochemistry, prognosis

## Abstract

TMPRSS11D (HAT) belongs to the large type II transmembrane serine protease (TTSP) family, participating in various biological and physiological processes. TMPRSS11D expression has been reported during squamous cell carcinogenesis, however, its expression during non-small cell lung cancer (NSCLC) development has not been studied. In this study, we determined the mRNA and protein expression of TMPRSS11D in NSCLC tumorous and matched adjacent normal tissues by quantitative reverse transcription PCR (qRT-PCR) and tissue microarray immunohistochemistry analysis (TMA-IHC) respectively. TMPRSS11D protein expression in tumorous tissues were correlated with NSCLC patients’ clinical characteristics and overall survival. Both TMPRSS11D mRNA and protein expression levels were significantly higher in NSCLC tumorous tissues than in adjacent normal tissues. High TMPRSS11D protein expression was associated with high TNM stages, and high TMPRSS11D protein expression is an independent prognostic marker in NSCLC. Based on our results, we conclude that TMPRSS11D could play a role in NSCLC development and progression. Because of its role in proteolysis of extracellular matrix, targeting TMPRSS11D may prevent the development of metastasis in NSCLC.

## INTRODUCTION

Lung cancer is the leading cause of cancer death worldwide and in China [1–4]. The estimated incidence and mortality of lung cancer in 2015 is 733.3 and 610.2 per 100,000 population respectively in China [5]. Lung cancer disproportionally affects men and urban populations than women and rural populations, and it is estimated that air pollution will be the primary cause of lung cancer in China by 2020 [6]. Majority lung cancers are non-small cell lung cancer (NSCLC) with following four histologic types: adenocarcinoma, squamous cell carcinoma, large cell carcinoma, and mixed histologies [7, 8]. Less than 50% of NSCLC patients are diagnosed with stage I or II disease, which are amendable for surgical resection with curative intent [9]. Even among patients who undergo such complete and presumably curative surgical treatment, approximately 40–50% of patients will have and die from recurrent disease [10]. Novel prognostic markers and therapeutic targets are needed to improve the overall survival of NSCLC patients [11].

TMPRSS11D belongs to the type II transmembrane serine protease (TTSP) family, which is the largest group of pericellular serine proteases. TTSP family is discovered through systematic genome-mining [12], containing a hydrophobic signal anchor at the N-terminal, an extracellular serine protease domain of the chymotrypsin (S1) fold at the C-terminal, and a variable stem region in the middle. The human TTSP family has 17 members, subdivided into four subfamilies. TMPRSS11D, also called human airway trypsin-like protease (HAT), belongs to the differentially expressed in squamous cell carcinoma (DESC) subfamily. It contains a single sea urchin sperm protein, enteropeptidase, agrin (SEA) domain in the middle.

TMPRSS11D was originally purified and cloned from the sputum of patients with chronic airway disease [13, 14]. It is highly expressed in respiratory epithelium, and its expression has also been detected in cervix, esophagus, prostate, trachea, and lung tissues [15]. Potential substrates of TMPRSS11D include fibrinogen [16], protease activated receptor (PAR)-2 [17], urokinase-type plasminogen activator receptor (uPAR, CD87) [18], and macrophage-stimulating protein (MSP) [19]. In addition, TMPRSS11D can proteolytically activate influenza A, influenza B, and severe acute respiratory syndrome coronavirus (SARS-CoV) [20–22], it is also expressed by important influenza and SARS-coronavirus target cells, therefore can support spread of these viruses in human host [23].

The role of TMPRSS11D in cancer has been investigated during squamous cell carcinogenesis [24]. TMPRSS11D protein is expressed on the surface of differentiated epithelial cells in normal cervical and esophageal epithelia, but significantly reduced or undetectable in late stages of cervical and esophageal cancers. Little is known about TMPRSS11D expression pattern in other types of cancer, including NSCLC.

In the current study, we measured both mRNA and protein level of TMPRSS11D in NSCLC tissue samples by quantitative reverse transcription PCR (qRT-PCR) and tissue microarray immunohistochemistry analysis (TMA-IHC) respectively. Subsequently, TMPRSS11D protein level was correlated with patients’ clinical characteristics and overall survival.

## RESULTS

### TMPRSS11D mRNA level was significantly higher in NSCLC tumorous tissues than in adjacent normal tissues

**Figure 1:**
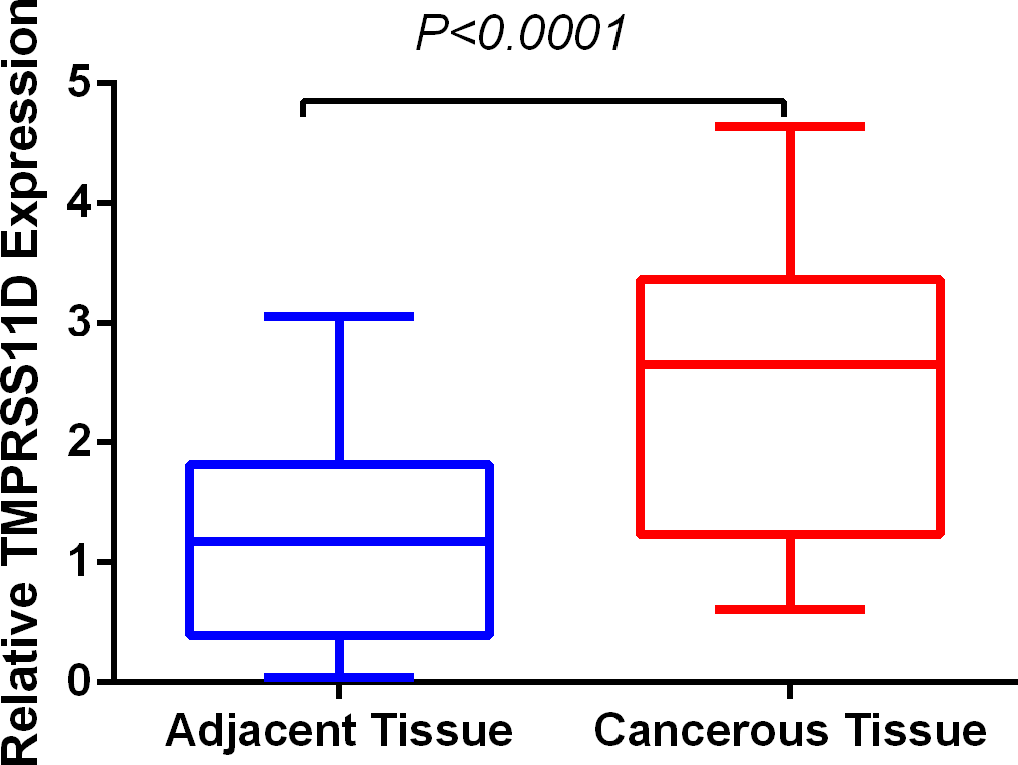
TMPRSS11D mRNA level was significantly higher in NSCLC tumorous tissues than in adjacent normal tissues TMPRSS11D mRNA was determined by qRT-PCR and relative quantification analysis by normalizing to GAPDH mRNA.

We determined TMPRSS11D mRNA level in 24 pairs of fresh frozen NSCLC tumorous and adjacent normal tissues. Relative TMPRSS11D mRNA expression level was normalized to the expression of housekeeping gene GAPDH. TMPRSS11D mRNA expression level was significantly higher in NSCLC tumorous tissues (2.451 ± 0.2481) when compared to adjacent normal tissues (1.174 ± 0.1625) (*P* < 0.001) (Figure 1).

### TMPRSS11D protein level was significantly higher in NSCLC tumorous tissues than adjacent normal tissues

**Figure 2:**
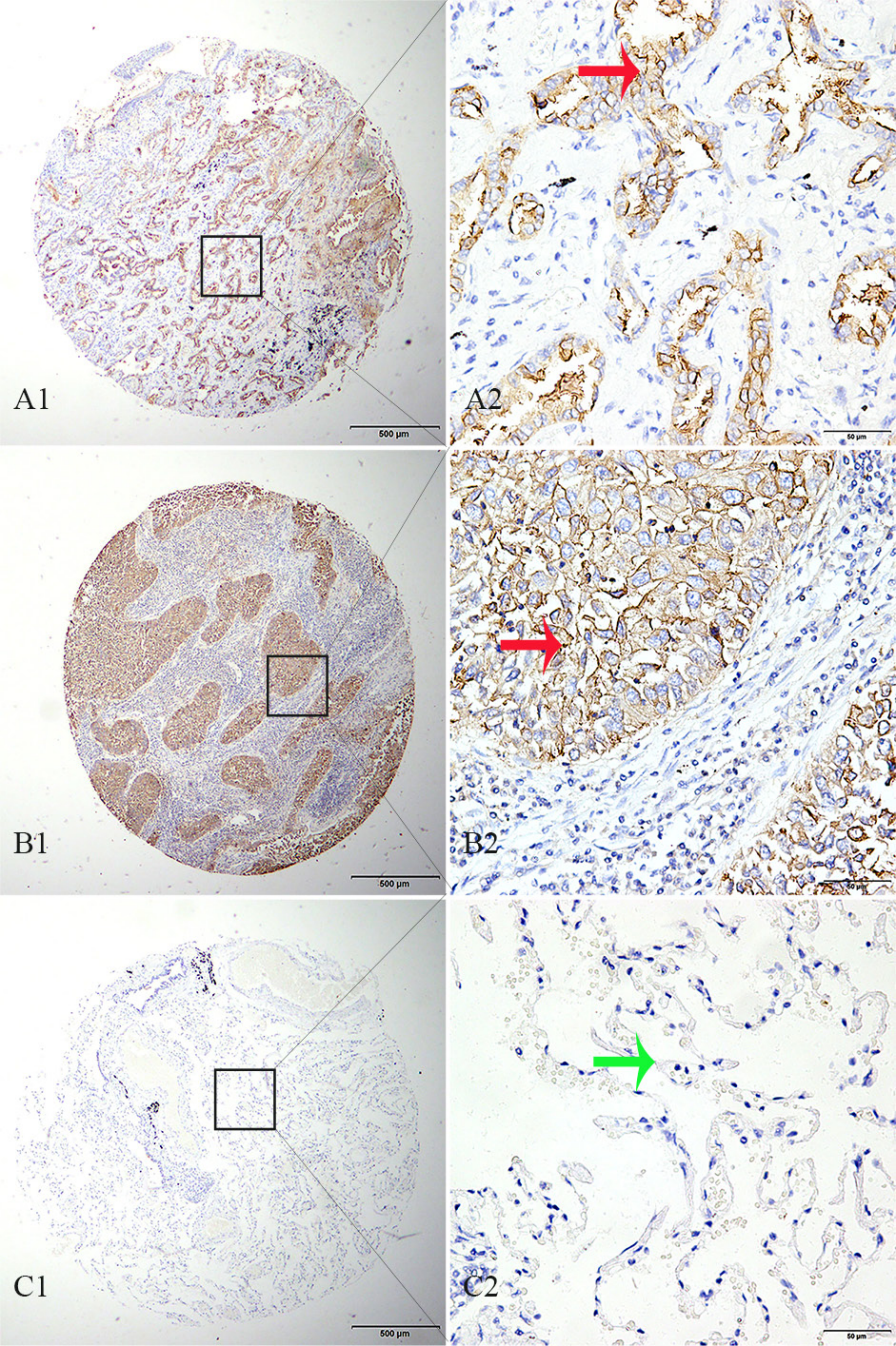
TMPRSS11D immunohistochemistry analysis in NSCLC and adjacent normal tissues **(A)** adenocarcinoma tissue strong positive for TMPRSS11D staining, **(B)** squamous cell carcinoma tissue strong positive for TMPRSS11D staining, **(C)** adjacent normal alveolar epithelium tissue negative for TMPRSS11D staining. A1-C1: 40× magnification (bar = 500 μm), A2–C2: 40× magnification (bar = 500 μm). Red arrow indicates positive TMPRSS11D staining, and green arrow indicates negative TMPRSS11D staining

We determined TMPRSS11D protein expression in 334 tumorous and 132 matched adjacent normal archived NSCLC tissue blocks. High TMPRSS11D expression was detected in 48.50% of tumorous tissues, significantly higher than 11.36% detected in normal lung tissues (Pearson χ^2^ = 55.399, *P* < 0.001, Figure 2).

### Association of TMPRSS11D expression with NSCLC clinical characteristics

Next, we correlated TMPRSS11D protein expression with NSCLC patients’ clinical characteristics, including gender, age at diagnosis, histological type, differentiation, and TNM stage. High TMPRSS11D protein expression was significantly associated with TNM staging (Pearson χ^2^ = 10.913, *P* = 0.004) (Table 1): present in 60.00% of stage III and IV patients, 57.14% of stage II patients, and 40.23% of stage 0 and I patients; as well as N stage (Pearson χ^2^ = 7.428, *P* = 0.024): present in 58.49% N2 stage patients, 58.11% N1 stage patients, and 42.86% N0 stage patients.

**Table 1:** Relationship between the expression of TMPRSS11D and clinicopathological characteristics in NSCLC

Characteristic	*n*	Low expression	High expression	Pearson χ^2^	*P*
Total	334	172 (51.50)	162 (48.50)		
Sex				0.941	0.332
Male	221	118 (53.39)	103 (46.61)		
Female	113	54 (47.79)	59 (52.21)		
Age				0.355	0.551
<60	187	99 (52.94)	88 (47.06)		
≥60	147	73 (49.66)	74 (50.34)		
Histological type				0.357	0.550
Adenocarcinoma	173	92 (53.18)	81 (46.82)		
SCC	111	55 (49.55)	56 (50.45)		
Others	50	25	25		
Differentiation				0.146	0.702
Low grade	90	49 (54.44)	41 (45.56)		
Middle and High grade	219	114 (52.05)	105 (47.95)		
Others	25	9	16		
TNM stage				10.913	0.004*
0–I	174	104 (59.77)	70 (40.23)		
II	91	39 (42.86)	52 (57.14)		
III + V	65	26 (40.00)	39 (60.00)		
Unknown	4	3	1		
T				1.400	0.497
Tis + T1	140	77 (55.00)	71 (45.00)		
T2	167	81 (48.50)	86 (51.50)		
T3,4	23	11 (47.83)	12 (52.17)		
Unknown	4	3	1		
N				7.428	0.024*
N0	203	116 (57.14)	87 (42.86)		
N1	74	31 (41.89) 950.00)	43 (58.11)		
N2	53	22 (41.51)	31 (58.49)		
Unknown	4	3	1		

* *p* < 0.05.

### High TMPRSS11D expression predicts poor overall survival in NSCLC patients

**Figure 3:**
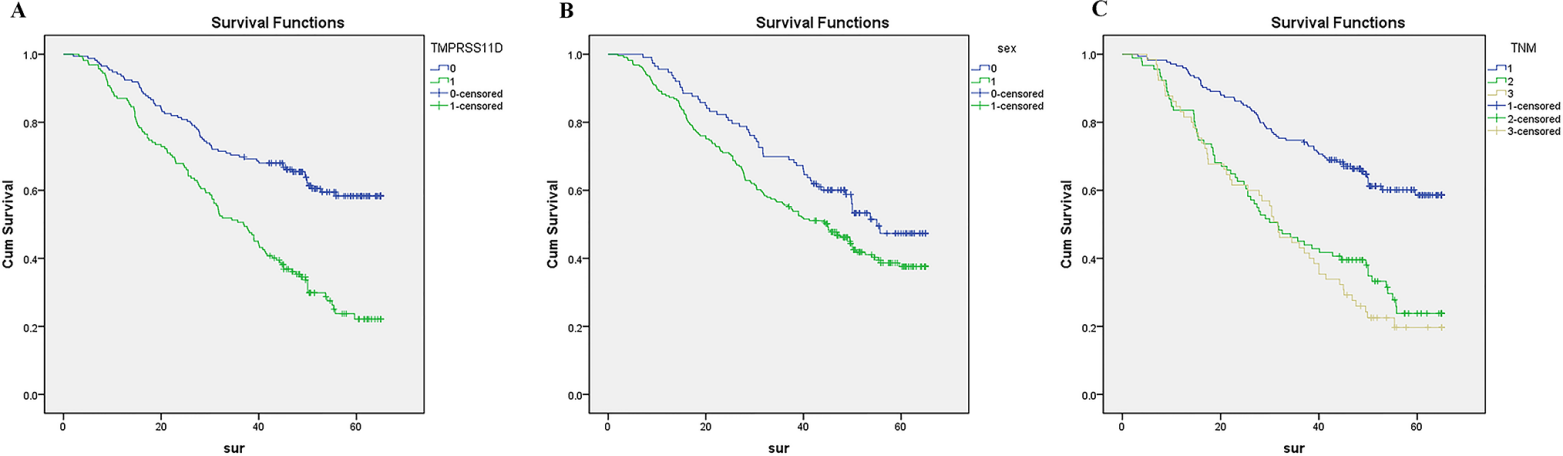
Survival curves of NSCLC patients by the Kaplan–Meier method and the log-rank test **(A)** NSCLC patients with high TMPRSS11D expression (green line, 1) had significantly worse overall survival than NSCLC patients with low or no TMPRSS11D expression (blue line, 0); **(B)** male NSCLC patients (green line, 1) had significantly worse overall survival than female NSCLC patients (blue line, 0); **(C)** stage III-IV NSCLC patients had worst overall survival (grey line, 3), than stage II NSCLC patients (green line, 2) and stage 0-I NSCLC patients (blue line, 1)

Finally, we analyzed prognostic factors in NSCLC patients using both univariate and multivariate analysis. In univariate analysis, high TMPRSS11D expression (HR, 2.412, 95% CI: 1.782–3.265; *P* < 0.001), sex (being male) (HR, 1.424, 95% CI: 1.034–1.960; *P* = 0.030), T stage (HR, 1.600, 95% CI: 1.261–2.030; *P* < 0.001), N stage (HR, 1.698, 95% CI: 1.428–2.018; *P* < 0.001), and TNM staging (HR, 1.755, 95% CI: 1.477–2.085; *P* < 0.001) were significantly associated with overall survival. TMPRSS11D expression, sex, and TNM staging were then included in the multivariate analysis. In multivariate analysis, high TMPRSS11D expression (HR, 2.246, 95% CI: 1.646–3.065; *P* < 0.001), sex (being male) (HR, 1.455, 95% CI: 1.055–2.007; *P* = 0.022), and TNM staging (HR, 1.617, 95% CI: 1.356–1.929; *P* < 0.001) remained significantly associated with poor overall survival (Table 2). Similar results were shown by the Kaplan-Meier survival curve (Figure 3).

**Table 2:** Univariate and multivariate analysis of different prognostic factors for 5-year survival in patients with NSCLC

Characteristic	Univariate analysis			Multivariate analysis		
	HR	*P*	95% CI	HR	*P*	95% CI
TMPRSS11D expression	2.412	< 0.001*	1.782–3.265	2.246	< 0.001*	1.646–3.065
High vs. low						
Age(years)	1.090	0.562	0.814–1.460			
≤ 60 vs. > 60						
Sex	1.424	0.030*	1.034–1.960	1.455	0.022*	1.055–2.007
Male vs. female						
Differentiation	0.867	0.395	0.625–1.204			
Well vs. moderate and poor						
Histological type	1.326	0.082	0.965–1.823			
Sq vs. Ad vs. others						
T	1.600	< 0.001*	1.261–2.030			
Tis + T1 vs. T2 vs. T3 + T4						
N	1.698	< 0.001*	1.428–2.018			
N0 vs. N1 vs. N2						
TNM stage	1.755	< 0.001*	1.477–2.085	1.617	< 0.001*	1.356–1.929
0–I vs II vs. III–IV						

* *p* < 0.05. TNM stage contains T stage and N stage, therefore, they were not included in the multivariate analysis.

## DISCUSSION

In the current study, we determined mRNA and protein expression levels of TMPRSS11D in both NSCLC tumorous and adjacent normal tissues. TMPRSS11D mRNA and protein level were significantly higher in tumorous tissues than in adjacent normal tissues. High TMPRSS11D protein level was significantly associated with TNM staging, and high TMPRSS11D protein expression is an independent prognostic marker for poor overall survival in NSCLC patients.

TMPRSS11D (HAT) belongs to the HAT/DESC subfamily of the type II transmembrane serine protease (TTSP) family, with additional four members in human genome: DESC1 (TMPRSS11E), TMPRSS1A, HATL4 (TMPRSS11F), and HATL5 (TMPRSS11B). Expression of DESC members are highly coordinated, and deletion in mice suggest that TMPRSS1A and TMPRSS11D are not essential for development, health, long-term survival [15]. TMPRSS11D protein is highly expressed in respiratory epithelium, localized in the suprabasal layer of bronchial epithelium as well as basal region of their associated cilia [25, 26]. Similarly TMPRSS11D protein is localized on the surface of differentiated epithelial cells in cervix and esophagus. Consistent with this observation, its expression was reduced or undetectable during cervical and esophageal cancer development, where epithelial cells undergo dedifferentiation [24]. Similar expression pattern was observed for DESC1 in head and neck cancer [27], and HATL5 (TMPRSS11B) in cervical, esophageal, head and neck cancer [28].

Unlike the cases in cervix and esophagus, we observed overexpression of TMPRSS11D in NSCLC compared to adjacent normal tissues, suggesting TMPRSS11D plays an oncogenic role in NSCLC development. This is consistent with the observation on other subfamily TTSP members. TMPRSS4 is overexpressed in pancreatic cancer when compared to healthy tissue. It is also highly expressed in thyroid, lung, liver, colon, ovarian, and gastric cancer [29]. It has been shown that overexpression of TMPRSS4 promotes invasion and metastasis through upregulating epithelial mesenchymal transition (EMT). TMPRSS2 is upregulated by androgenic hormone in prostate cancer cells [30]. In addition, TMPRSS2-ERG fusion is present in 40%–80% of prostate cancers, overexpression of estrogen-regulated gene (ERG) due to gene fusion is associated with a more aggressive phenotype in prostate cancer [31, 32].

Mechanistically, based on its known substrates, TMPRSS11D can promote tumorigenesis through cell proliferation, invasion and metastasis, and inflammation. Through PAR-2, TMPRSS11D can increase intracellular free Ca2+ [33], and stimulate proliferation of human bronchial fibroblast cells through MEK-MAPK signaling pathway [17]. Through MSP, TMPRSS11D can activate the MSP-RON signaling pathway in the respiratory tract [19], a signaling pathway involved in invasive growth of different types of cancer [34]. Through PAR-2, TMPRSS11D can mediate IL-8 production and accumulation of inflammatory cells [35]; through amphiregulin (AR), TMPRSS11D can activate regulatory T lymphocytes and suppresses CD8+ T cell-mediated antitumor response [36]. Alternatively, TMPRSS11D might have its unique substrates during NSCLC development. Identification of TMPRSS11D substrates and inhibitors in NSCLC is an essential step towards the development of novel therapies based on TMPRSS11D.

Our study suffers the same limitations of retrospective studies: our results might be subject to sample selection bias, extension to other populations needs further validation. For example, our data showed that male NSCLC patients had poor overall survival than female NSCLC patients, currently, we do not know whether this reflects the biology of NSCLC or this is due to sample selection bias; IHC data are semi-quantitative, therefore, our results should be validated using alternative methods; we did not determine the expression of other HAT/DESC subfamily members in NSCLC, though it has been reported that these genes are coordinately expressed and may be functionally redundant. We did not perform *in vitro* functional analysis, thus could not determine whether TMPRSS11D overexpression is the cause or the consequence of NSCLC progression.

In conclusion, our study demonstrates that TMPRSS11D could play an important role in the development of NSCLC and TMPRSS11D overexpression is an independent prognostic marker for NSCLC in Chinese population. Future clinical studies should investigate the role of other HAT/DESC subfamily members in NSCLC, and *in vitro* functional studies are needed to decipher the differential roles TMPRSS11D plays in various types of cancer. These studies are necessary to determine whether HAT/DESC family members are potential novel therapeutic targets in NSCLC.

## MATERIALS AND METHODS

### Human tissue specimens and patient clinical information

A total of 358 NSCLC patients were included in the study. Twenty four NSCLC patients were consented and enrolled before surgery during 2014–2015, and 24 pairs matched tumorous and normal fresh tissue samples were collected and frozen at the time of surgery. There were 15 men and 9 women, 7 squamous cell carcinoma and 17 adenocarcinoma. in the 24 pairs of samples. In addition, 334 NSCLC patients provided 334 cancerous tissues, 132 matched adjacent normal tissues formalin-fixed paraffin-embedded (FFPE) tissue blocks from 2009–2010. At the time of surgery, patients’ age ranged from 30 to 81 years, with a median of 61.3 years. Other details were shown in Table 1. Clinical characteristics were obtained from patients’ medical records. The study protocol was approved by the Human Research Ethics Committee of the Affiliated Hospital of Nantong University, Jiangsu, China.

### TMPRSS11D mRNA and protein expression and statistical analysis

TMPRSS11D mRNA level was determined by quantitative reverse transcription PCR (qRT-PCR) using relative quantification method by normalizing to the house keeping gene GAPDH [37]. The primers used are as follows: TMPRSS11D forward primer (5′- TAC ACAGGAATACAGGACTT-3′) and TMPRSS11D reverse primer (5′- CTCACACCACTACCATCT-3′), GAPDH forward primer (5′-TGC ACC ACC AAC TGC TTA GC-3′) and GAPDH reverse primer (5′-GGC ATG GAC TGT GGT CAT GAG-3′). TMPRSS11D protein expression in tissue blocks was determined using tissue microarray immunohistochemistry (TMA IHC). Rabbit polyclonal anti-human TMPRSS11D antibody was used (dilution 1:200, ab127031, Abcam, USA). The TMPRSS11D IHC data were scored using the semi-quantitative H-score method taking into account both the staining intensity and the percentage of cells at that intensity [38] ranging from 0–300. Subsequently, the continuous TMPRSS11D protein expression data were converted into dichotic data (low vs high) using specific cutoff values, which were selected to be significant in terms of overall survival (OS) using the X-tile software program (The Rimm Lab at Yale University; http://medicine.yale.edu/lab/rimm/research/software.aspx) [39, 40]. In the current study, the cutoff was 100: score 0–100 was considered low TMPRSS11D expression while 101–300 was considered high TMPRSS11D expression.

Statistical analysis was performed as described before [41]. Student *t* test was used to compare TMPRSS11D mRNA and protein expression between tumorous and normal tissue samples. Pearson χ^2^ tests were performed to determine the correlation between TMPRSS11D expression and clinicopathologic parameters. Univariate and multivariate Cox regression models were used to identify prognostic factors. Kaplan-Meier method was used to calculate survival curves. For all analyses, a *P*-value < 0.05 was regarded as statistically significant. Data were analyzed using SPSS 20.0 statistics software (SPSS Inc., Chicago, IL, USA) and STATA 12.0 (Stata Corp, College Station, TX, USA).

## References

[1] Hong QY, Wu GM, Qian GS, Hu CP, Zhou JY, Chen LA, Li WM, Li SY, Wang K, Wang Q, Zhang XJ, Li J, Gong X (2015). Prevention and management of lung cancer in China. Cancer.

[2] Fitzmaurice C, Dicker D, Pain A, Hamavid H, Moradi-Lakeh M, MacIntyre MF, Allen C, Hansen G, Woodbrook R, Wolfe C, Hamadeh RR, Moore A, Werdecker A (2015). The Global Burden of Cancer 2013. JAMA Oncol.

[3] Zhou C (2014). Lung cancer molecular epidemiology in China: recent trends. Transl Lung Cancer Res.

[4] Ferlay J, Soerjomataram I, Dikshit R, Eser S, Mathers C, Rebelo M, Parkin DM, Forman D, Bray F (2015). Cancer incidence and mortality worldwide: sources, methods and major patterns in GLOBOCAN 2012. Int J Cancer.

[5] Chen W, Zheng R, Baade PD, Zhang S, Zeng H, Bray F, Jemal A, Yu XQ, He J (2016). Cancer statistics in China, 2015. CA Cancer J Clin.

[6] Chen W, Zheng R, Zeng H, Zhang S (2015). Epidemiology of lung cancer in China. Thorac Cancer.

[7] Antonelli G, Libra M, Panebianco V, Russo AE, Vitale FV, Colina P, D'Angelo A, Rossello R, Ferrau F (2016). Molecular-targeted therapy for elderly patients with advanced non-small cell lung cancer. Oncol Lett.

[8] Ho C, Tong KM, Ramsden K, Ionescu DN, Laskin J (2015). Histologic classification of non-small-cell lung cancer over time: reducing the rates of not-otherwise-specified. Curr Oncol.

[9] McElnay P, Lim E (2014). Adjuvant or neoadjuvant chemotherapy for NSCLC. J Thorac Dis.

[10] Padda SK, Burt BM, Trakul N, Wakelee HA (2014). Early-stage non-small cell lung cancer: surgery, stereotactic radiosurgery, and individualized adjuvant therapy. Semin Oncol.

[11] Burotto M, Thomas A, Subramaniam D, Giaccone G, Rajan A (2014). Biomarkers in early-stage non-small-cell lung cancer: current concepts and future directions. J Thorac Oncol.

[12] Bugge TH, Antalis TM, Wu Q (2009). Type II transmembrane serine proteases. J Biol Chem.

[13] Yasuoka S, Ohnishi T, Kawano S, Tsuchihashi S, Ogawara M, Masuda K, Yamaoka K, Takahashi M, Sano T (1997). Purification, characterization, and localization of a novel trypsin-like protease found in the human airway. Am J Respir Cell Mol Biol.

[14] Yamaoka K, Masuda K, Ogawa H, Takagi K, Umemoto N, Yasuoka S (1998). Cloning and characterization of the cDNA for human airway trypsin-like protease. J Biol Chem.

[15] Sales KU, Hobson JP, Wagenaar-Miller R, Szabo R, Rasmussen AL, Bey A, Shah MF, Molinolo AA, Bugge TH (2011). Expression and genetic loss of function analysis of the HAT/DESC cluster proteases TMPRSS11A and HAT. PLoS One.

[16] Yoshinaga S, Nakahori Y, Yasuoka S (1998). Fibrinogenolytic activity of a novel trypsin-like enzyme found in human airway. J Med Invest.

[17] Matsushima R, Takahashi A, Nakaya Y, Maezawa H, Miki M, Nakamura Y, Ohgushi F, Yasuoka S (2006). Human airway trypsin-like protease stimulates human bronchial fibroblast proliferation in a protease-activated receptor-2-dependent pathway. Am J Physiol Lung Cell Mol Physiol.

[18] Beaufort N, Leduc D, Eguchi H, Mengele K, Hellmann D, Masegi T, Kamimura T, Yasuoka S, Fend F, Chignard M, Pidard D (2007). The human airway trypsin-like protease modulates the urokinase receptor (uPAR, CD87) structure and functions. Am J Physiol Lung Cell Mol Physiol.

[19] Orikawa H, Kawaguchi M, Baba T, Yorita K, Sakoda S, Kataoka H (2012). Activation of macrophage-stimulating protein by human airway trypsin-like protease. FEBS Lett.

[20] Baron J, Tarnow C, Mayoli-Nussle D, Schilling E, Meyer D, Hammami M, Schwalm F, Steinmetzer T, Guan Y, Garten W, Klenk HD, Matriptase Bottcher-Friebertshauser E. (2013). HAT, and TMPRSS2 activate the hemagglutinin of H9N2 influenza A viruses. J Virol.

[21] Bottcher-Friebertshauser E, Klenk HD, Garten W (2013). Activation of influenza viruses by proteases from host cells and bacteria in the human airway epithelium. Pathog Dis.

[22] Bertram S, Glowacka I, Muller MA, Lavender H, Gnirss K, Nehlmeier I, Niemeyer D, He Y, Simmons G, Drosten C, Soilleux EJ, Jahn O, Steffen I (2011). Cleavage and activation of the severe acute respiratory syndrome coronavirus spike protein by human airway trypsin-like protease. J Virol.

[23] Bertram S, Heurich A, Lavender H, Gierer S, Danisch S, Perin P, Lucas JM, Nelson PS, Pohlmann S, Soilleux EJ (2012). Influenza and SARS-coronavirus activating proteases TMPRSS2 and HAT are expressed at multiple sites in human respiratory and gastrointestinal tracts. PLoS One.

[24] Duhaime MJ, Page KO, Varela FA, Murray AS, Silverman ME, Zoratti GL, List K (2016). Cell Surface Human Airway Trypsin-Like Protease Is Lost During Squamous Cell Carcinogenesis. J Cell Physiol.

[25] Chokki M, Yamamura S, Eguchi H, Masegi T, Horiuchi H, Tanabe H, Kamimura T, Yasuoka S (2004). Human airway trypsin-like protease increases mucin gene expression in airway epithelial cells. Am J Respir Cell Mol Biol.

[26] Takahashi M, Sano T, Yamaoka K, Kamimura T, Umemoto N, Nishitani H, Yasuoka S (2001). Localization of human airway trypsin-like protease in the airway: an immunohistochemical study. Histochem Cell Biol.

[27] Sedghizadeh PP, Mallery SR, Thompson SJ, Kresty L, Beck FM, Parkinson EK, Biancamano J, Lang JC (2006). Expression of the serine protease DESC1 correlates directly with normal keratinocyte differentiation and inversely with head and neck squamous cell carcinoma progression. Head Neck.

[28] Miller GS, Zoratti GL, Murray AS, Bergum C, Tanabe LM, List K (2014). HATL5: a cell surface serine protease differentially expressed in epithelial cancers. PLoS One.

[29] Ohler A, Becker-Pauly C (2012). TMPRSS4 is a type II transmembrane serine protease involved in cancer and viral infections. Biol Chem.

[30] Lin B, Ferguson C, White JT, Wang S, Vessella R, True LD, Hood L, Nelson PS (1999). Prostate-localized and androgen-regulated expression of the membrane-bound serine protease TMPRSS2. Cancer Res.

[31] Demichelis F, Fall K, Perner S, Andren O, Schmidt F, Setlur SR, Hoshida Y, Mosquera JM, Pawitan Y, Lee C, Adami HO, Mucci LA, Kantoff PW (2007). TMPRSS2: ERG gene fusion associated with lethal prostate cancer in a watchful waiting cohort. Oncogene.

[32] Guo XQ, Gui YT, Cai ZM (2011). [The progress of TMPRSS2-ETS gene fusions and their mechanism in prostate cancer]. Yi Chuan.

[33] Miki M, Nakamura Y, Takahashi A, Nakaya Y, Eguchi H, Masegi T, Yoneda K, Yasuoka S, Sone S (2003). Effect of human airway trypsin-like protease on intracellular free Ca2+ concentration in human bronchial epithelial cells. J Med Invest.

[34] Yao HP, Zhou YQ, Zhang R, Wang MH (2013). MSP-RON signalling in cancer: pathogenesis and therapeutic potential. Nat Rev Cancer.

[35] Iwakiri K, Ghazizadeh M, Jin E, Fujiwara M, Takemura T, Takezaki S, Kawana S, Yasuoka S, Kawanami O (2004). Human airway trypsin-like protease induces PAR-2-mediated IL-8 release in psoriasis vulgaris. J Invest Dermatol.

[36] Yuan CH, Sun XM, Zhu CL, Liu SP, Wu L, Chen H, Feng MH, Wu K, Wang FB (2015). Amphiregulin activates regulatory T lymphocytes and suppresses CD8+ T cell-mediated anti-tumor response in hepatocellular carcinoma cells. Oncotarget.

[37] Zhang HJ, Yao DF, Yao M, Huang H, Wang L, Yan MJ, Yan XD, Gu X, Wu W, Lu SL (2013). Annexin A2 silencing inhibits invasion, migration, and tumorigenic potential of hepatoma cells. World J Gastroenterol.

[38] Detre S, Saclani Jotti G, Dowsett M (1995). A “quickscore” method for immunohistochemical semiquantitation: validation for oestrogen receptor in breast carcinomas. J Clin Pathol.

[39] Huang J, Fan X, Wang X, Lu Y, Zhu H, Wang W, Zhang S, Wang Z (2015). High ROR2 expression in tumor cells and stroma is correlated with poor prognosis in pancreatic ductal adenocarcinoma. Sci Rep.

[40] Lu C, Wang X, Zhu H, Feng J, Ni S, Huang J (2015). Over-expression of ROR2 and Wnt5a cooperatively correlates with unfavorable prognosis in patients with non-small cell lung cancer. Oncotarget.

[41] Liu X, Xu Y, Jin Q, Wang W, Zhang S, Wang X, Zhang Y, Xu X, Huang J (2016). EphA8 is a prognostic marker for epithelial ovarian cancer. Oncotarget.

